# Economic policy – Public health linkage and the importance of a regional platform: The case of tobacco control

**DOI:** 10.1177/1468018115600123f

**Published:** 2015-12

**Authors:** Jenina Joy Chavez

**Affiliations:** Action for Economic Reforms - Industrial Policy Team, Philippines

Southeast Asia has been the source of some recent encouraging developments on the health front, particularly in the area of tobacco control. In December 2012, the Philippines passed a landmark sin tax legislation that restructures the decade-old tax system for tobacco and alcohol products, updates the tax rates and indexes them for automatic increases, and earmarks its incremental proceeds for universal health financing. In August 2013, Malaysia proposed to exclude tobacco from the Trans-Pacific Partnership Agreement (TPPA), a bid to protect tobacco control measures from any state-to-state or investor–state challenge. Earlier in July 2012, it was reported that Health Ministers of the Association of Southeast Asian Nations (ASEAN) agreed to withdraw tobacco from the ASEAN Free Trade Agreement (AFTA). While their actual Joint Statement made no mention of trade, the language referring to tobacco control was clear (ASEAN, 2012).

Of foremost interest in these developments is the increasing awareness of the linkages between public health regulation and economic policy. Health arguments are being advanced to affect economic policy, with the ultimate objective of reorienting incentives to improve health outcomes. Specifically, tax, trade and investment policies are being targeted. However, the likely impact on tobacco control may not be as straightforward.

There are two ways in which trade and investment agreements can be bad for tobacco control. One, when trade liberalisation or the lowering of tariffs leads to more competition that results in cheaper products that can trigger more consumption. Two, when clauses like investor protection and intellectual property protection in these agreements place limits on a country’s capacity to regulate.

In the case of ASEAN countries, a study by Drope and Chavez (2015) has shown that the impact on affordability and consumption of trade liberalisation has been mixed. Where liberalisation has been deepest (Malaysia), cigarette prices have become less affordable and consumption has been on the decline, while the least liberalised and a state monopoly, to boot (Vietnam), has experienced fluctuations in affordability and increased consumption. Given this, it is yet unclear whether the total exclusion of tobacco in trade agreements like AFTA is the most effective way of curbing tobacco use.

On the other hand, new generation free trade agreements (FTAs) contain investment chapters that provide very liberal protection to investments – including the ability to lodge claims against a country in international arbitration not governed by domestic law. The TPPA, of which Brunei, Singapore, Malaysia and Vietnam are a part, and where others like the Philippines intend to join, is a potential red flag as it could provide broader investor protection than is already contained in many current arrangements. That said, these countries already have existing agreements with countries where big tobacco corporations are based (for instance, Switzerland – Philip Morris International/PMI and Japan Tobacco International, and the United Kingdom – British American Tobacco/BAT), so even sans the newer more ambitious FTAs, the potential risks already exist. Therefore, a specific carveout of tobacco from new agreements will have to consider existing ones as well.

On the issue of foreign direct investment (FDI), most low- and middle-income countries are in the business of attracting FDI as a development strategy. To induce these FDIs, various incentives in the form of tax holidays, subsidies and privileges are provided. All this helps to lower production costs and ultimately product prices, and affect increases in demand. The Framework Convention on Tobacco Control (FCTC Article 5.3) (WHO 2003) explicitly proscribes the giving of incentives to the tobacco industry.

Transnational tobacco companies have invested substantially in most Southeast Asian countries even before open trade, the objective being to establish strategic presence, a trend that continues even after the period of liberalisation (Drope and Chavez, 2015). In certain cases, like in the Philippines (where PMI invested in a manufacturing plant in 2000 and 2007), these investments, while located in special protected zones, were made without any incentives asked for or granted, highlighting the priority placed on getting domestic market space (Chavez et al., 2014; Drope et al., 2014).

The Philippine sin tax example provides interesting insights into the political dynamics of FDI. In the 1990s and 2000s, PMI was among the biggest supporters of sin tax reform. This support was part of the attempt to break the stranglehold of Fortune Tobacco, with the tax reform expected to introduce more competition by applying non-discriminatory rates between local and imported brands. In 2010, PMI and FTC created a joint venture, the Philip Morris Fortune Tobacco Corporation (PMFTC), which gave PMI a foothold in the local market. When the sin tax campaign was revived, PMI through PMFTC became the most rabid opposition. BAT, trying to enter the market, was the only industry player supportive of the reform. Clearly, for FDI market entry is primary, but once in, FDI will protect its business to the full extent.

Taxation is considered the most effective tobacco control measure in terms of affecting prices and affordability, and in turn reducing consumption and the prevalence of smoking (WHO, 2015). However, it is highly unpopular and difficult to introduce. In the region, Malaysia and Singapore have been known for substantial increases in their tobacco taxes by 2010 (Drope and Chavez, 2015). The Philippine sin tax reform met success through the earmarking of the revenue to universal health to generate public support. It was also a successful coming-together of two sets of policy reform advocates – on public health, including tobacco control, and economic reforms, specifically tax reform (Chavez et al., 2014).

For sure, the tobacco industry remains a powerful political and economic actor in the region. The experience in the region shows not just the difficulty of instituting tobacco control measures, but also the complexity of issues implied in them. At the same time, the public health challenge of tobacco use is immense. One in five ASEAN citizens smoke; more than 500 Indonesian deaths daily are accounted for by smoking; tobacco-related healthcare costs can be as high as US$1.78 billion for Indonesia; and both youth smoking and youth intention to smoke are high (Southeast Asia Tobacco Control Alliance [SEATCA], 2014).

The complex linkage of public health and economic policy is evident in the way countries respond to communicable diseases, like MERS, SARS and HIV-AIDS, among others. Countries’ capacity to deal with these diseases is often linked to the impact on economic productivity, and health costs. In the context of freer mobility and greater regional connectivity, disease spread across borders and how it affects travel, tourism and the regional economy becomes an equally legitimate concern.

To ASEAN’s (2014) credit, NCDs are likewise given a prominent emphasis in line with ‘health promotion, and healthy lifestyle’. It is most aggressive in tobacco control, having identified Focal Points on Tobacco Control and making direct references to FCTC (ASEAN, 2013). The consideration of tobacco exclusion from AFTA is the boldest statement made on tobacco control thus far.

Unfortunately, the increased activism of ASEAN health officials on tobacco control is undermined by their lack of mandate on economic issues. Trade is the domain of economic ministers; hence, health ministers calling for the withdrawal of tobacco from AFTA is more a symbolic rather than a binding move. Moreover, there had been no discussion between the two sets of ministers to find ways to pursue it in the agenda.

The vital role of ASEAN cannot be overemphasised. Its significance does not stop with the negotiation and signing of various trade and economic agreements, but extends to the need to ensure that other development goals are met. Better understanding of existing economic policy is needed to inform public health measures. More importantly, there is need to link and harmonise public health and economic objectives. For this, better coordination and cooperation among different policymakers and stakeholders are crucial.
